# Low-Dose Metformin Treatment Reduces In Vitro Growth of the LL/2 Non-small Cell Lung Cancer Cell Line

**DOI:** 10.3390/biomedicines11010065

**Published:** 2022-12-27

**Authors:** Nicole L. Stott Bond, Didier Dréau, Ian Marriott, Jeanette M. Bennett, Michael J. Turner, Susan T. Arthur, Joseph S. Marino

**Affiliations:** 1 Distance Education, Technology and Integration, University of North Georgia, Dahlonega, GA 30597, USA; 2 Department of Biological Sciences, University of North Carolina at Charlotte, Charlotte, NC 28223, USA; 3 Department of Psychological Science, University of North Carolina at Charlotte, Charlotte, NC 28223, USA; 4 Laboratory of Systems Physiology, Department of Applied Physiology, Health, and Clinical Sciences, University of North Carolina at Charlotte, Charlotte, NC 28223, USA

**Keywords:** non-small cell lung cancer, metformin, cancer cell proliferation

## Abstract

Lung cancer maintains a relatively small survival rate (~19%) over a 5-year period and up to 80–85% of all lung cancer diagnoses are Non-Small Cell Lung Cancer (NSCLC). To determine whether metformin reduces non-small cell lung cancer (NSCLC) LL/2 cell growth, cells were grown in vitro and treated with metformin for 48 h. qPCR was used to assess genes related to cell cycle regulation and pro-apoptotic markers, namely Cyclin D, CDK4, p27, p21, and HES1. Treatment with 10 mM metformin significantly reduced HES1 expression (*p* = 0.011). Furthermore, 10 mM metformin treatment significantly decreased REDD1 (*p* = 0.0082) and increased p-mTOR Ser2448 (*p* = 0.003) protein expression. Control cells showed significant reductions in phosphorylated p53 protein expression (*p* = 0.0367), whereas metformin treated cells exhibited reduced total p53 protein expression (*p* = 0.0078). There were no significant reductions in AMPK, PKB/AKT, or STAT3. In addition, NSCLC cells were treated for 48 h. with 10 mM metformin, 4 µM gamma-secretase inhibitor (GSI), or the combination of metformin (10 mM) and GSI (4 µM) to determine the contribution of respective signaling pathways. Metformin treatment significantly reduced total nucleus expression of the proliferation maker Ki-67 with an above 65% reduction in Ki-67 expression between control and metformin-treated cells (*p* = 0.0021). GSI (4 µM) treatment significantly reduced Ki-67 expression by ~20% over 48 h (*p* = 0.0028). Combination treatment (10 mM metformin and 4 µM GSI) significantly reduced Ki-67 expression by more than 50% over 48 h (*p* = 0.0245). As such, direct administration of metformin (10 mM for 48 h) proved to be an effective pharmaceutical agent in reducing the proliferation of cultured non-small cell cancer cells. These intriguing in vitro results, therefore, support the further study of metformin in appropriate in vivo models as an anti-oncogenic agent and/or an adjunctive therapy.

## 1. Introduction

In the United States, lung cancer remains the second-most-common type of cancer and represents approximately 13% of all new cancer cases (SEER, National Cancer Institute). In 2022, lung cancer contributed to approximately 130,180 fatalities, implying more than 350 fatalities daily [1]. By 2030, the yearly diagnoses are expected to reach 225,000 in the United States alone [2]. Non-small cell lung cancer (NSCLC) accounts for 80–85% of all new lung cancer diagnoses [3]. The relative survival rate of lung cancer patients remains very low, with an average 19% 5-year survival rate (15% for men and 22% for women). Both research and medicine are making significant strides to improve treatment modalities for cancer patients; however, the prevalence and severity of lung cancer requires continued refinement to improve patient outcomes and minimize systemic complications. 

Metformin, a commonly used and well-tolerated type 2 diabetic drug, is widely used to promote glucose uptake, increase insulin sensitivity, and improve systemic glucose metabolism [4,5]. Metformin primarily acts through activating adenosine monophosphate (AMPK) activated protein kinase (AMPK). AMPK is an important cellular energy sensor that promotes insulin sensitivity in peripheral tissues and reduces adipocyte formation [4]. Importantly, metformin elicits anti-tumorigenic effects in many cancers, including prostate, colon, breast, skin, and obesity-activated thyroid cancer [6,7,8,9,10]. In cancers, metformin induces alterations in cellular proliferation, apoptosis, cell cycle progression, and inflammatory responses through signaling pathways including AMPK, mechanistic target of rapamycin (mTOR), mitogen-activated protein kinase (MAPK), nuclear factor kappa-light-chain-enhancer of activated B cells (NF-kB), and signal transducer and activator of transcription 3 (STAT3) pathways [11,12,13,14]. 

Because metformin canonically inhibits the electron transport chain, numerous cancer studies demonstrate that metformin’s anti-cancer activities involve AMPK- dependent mechanisms. When mitochondrial function is impaired, liver kinase B 1 (LKB1) phosphorylates Threonine 172, activating AMPK [15]. LKB-1-activation of AMPK phosphorylates and activates Tumor sclerosis complex 1 and 2 (TSC 1/2), leading to a negative regulation of mTOR activity, reducing cell growth and proliferation [16]. Activated AMPK also induces cell cycle arrest through p53 activation leading to upregulation of pro-apoptotic genes [17,18]. 

Metformin also combats tumorigenesis independently of AMPK through reduced insulin like growth factor (IGF-1) signaling. Concomitant with insulin and IGF-,1 phosphatidylinositol-3,4,5-triphosphate (PIP3) recruitment occurs, leading to subsequent activation of Protein Kinase B/AKT [19,20]. AKT phosphorylates TSC1/2, rendering TSC1/2 inactive and increasing mTOR activity [21]. Indeed, in a Lewis lung carcinoma mouse model, the expression of fundamental genes involved in the phosphatidylinositol 3-kinase (PI3K)–protein kinase B (Akt) pathway were attenuated [22]. The PI3K/AKT pathway, which is often constitutively active in tumor cells, plays an important role in cellular proliferation, growth, metabolism, and protein synthesis [23]. Reduced expression of regulatory genes in the PI3K/AKT pathway could lead to mitochondrial dysfunction [22].

Notch signaling is a well-conserved transmembrane cellular signaling pathway particularly important for determining cell fate, both during development and in maintaining homeostasis of adult tissues [24]. Notch is a ligand-dependent intercellular pathway regulating cellular differentiation, proliferation, and apoptosis. Activation of Notch signaling occurs when ubiquitinated ligands (jagged or delta-like proteins) initiate a cleavage cascade via metalloproteases and γ-secretases, releasing the Notch intracellular domain (NCID) from the transmembrane domain [25]. NCID released into the cytosol then translocates to the nucleus, leading to the transcription of target genes, including hair and enhancer of split 1 (HES1). Alterations in Notch signaling, particularly over-activation, are prevalent in many diseases, including cancer [26]. Over-activation of Notch signaling drives Non-Small Cell Lung Cancer (NSCLC) tumorigenesis [27], and other NSCLC studies have found mutations within the Notch signaling cascade [28]. 

The AMPK-independent action of metformin on NSCLC progression remains largely uncharacterized, possibly because of metformin’s primary role as an AMPK activator. While metformin demonstrates antineoplastic effects via cell cycle arrest, the mechanism underlying its AMPK-independent pharmaceutical’s action on NSCLC tumor development still requires elucidation. The purpose of this study was to better understand the oncogenic programming alternations metformin elicits on NSCLC cell proliferation.

## 2. Materials and Methods

### 2.1. Cell Culture

Non-small cell lung cancer (NSCLC) cells (Cl050 https://imanislife.com/products/ll2-fluc-puro/; accessed on 18 July 2018, Imanis Life Sciences, Rochester, MN, USA) were grown in Dulbecco’s Modified Eagle Medium (DMEM) supplemented with 10% fetal bovine serum and 1% penicillin–streptomycin. Cells were passaged in 2 µg/mL puromycin to maintain high luciferase fluorescence and cell passages two and six were utilized for all experiments. Cells were maintained at 37 °C for 48 h or until predetermined time points.

### 2.2. Metformin Treatment

NSCLC cells were seeded at a density of 30,000 cells/well and allowed to proliferate for 24 h (~30% confluence). Cells were treated with varying isovolumetric concentrations (5 mM–15 mM) of metformin hydrochloride (PHR1084; Sigma, St. Louis, MO, USA) for pre-determined time points to assess the optimal treatment concentration. 

### 2.3. Gamma Secretase Inhibition

NSCLC were seeded at a density of 15,000 cells/well and allowed to proliferate for 24 h (~30% confluence). A gamma secretase inhibitor (565771; Sigma) was added to each well at a final concentration of 4 uM GSI/well (control cells received equal volume DMSO) every 12 h for 48 h. Cells were collected for both mRNA analyses and immunocytochemistry. 

### 2.4. Co-Treatment with Metformin and Gamma Secretase Inhibition

NSCLC were seeded at a density of 15,000 cells/well and allowed to proliferate for 24 h (~30% confluence). A gamma secretase inhibitor (565771; Sigma) was added to each well at a final concentration of 4 uM GSI/well (control cells received equal volume DMSO) every 12 h for 48 h. Metformin hydrochloride was added to wells at a final of 10 mM Mmetformin/well. Cells were analyzed via immunofluorescence. 

### 2.5. MTT Assay

NSCLC cells were grown for 24 h (~30% confluence) and serum-starved overnight with serum-free media to induce quiescence. Cells were treated with equal volumes of fresh growth media followed by metformin treatment with a range of concentrations from 5 mM to 15 mM. NSCLC cell viability was assessed every 24 h for 3 days via an MTT assay using 3-(4,5-dimethyltiazol-2-yl)-2,5-diphenyltetrazolium bromide (MTT) reagent (M2128; Sigma). MTT reagent was added to wells at a final concentration of 1 mg/mL 4 h prior to the collection time point, allowing for the formation of blue formazan crystals in growing cells. Crystals were dissolved with dimethyl sulfoxide (DMSO, D8418; Sigma). Absorbance was measured at 560 nm.

### 2.6. Cell Lysate Homogenization and mRNA Quantification

mRNA was extracted utilizing a GeneJET RNA purification kit (K0731; Thermo Fisher Scientific, Waltham, MA, USA). Briefly, growth media was removed from adherent cells; cells were washed with 1x PBS, and removed from the culture plate by scraping in 50 µL 1x PBS. Cells were transferred to a microcentrifuge tube and centrifuged for 5 min at 250× *g*. Supernatant was removed, and cells were resuspended in lysis buffer supplemented with 2% β-mercaptoethanol and gently vortexed for 10 s. A total of 360 µL of ethanol (96–100%) was added to precipitate the mRNA. The cell lysate was transferred to a GeneJET purification column and centrifuged for 1 min at 12,000× *g*. After all lysate was filtered through the column, 700 µL of Wash Buffer 1 (supplemented with 20% ethanol) was added to the column and centrifuged for 1 min at 12,000× *g*. Next, 600 µL of Wash Buffer 2 (supplemented with 63% ethanol) was added to the column and centrifuged for 1 min at 12,000× *g*. A total of 250 µL of Wash Buffer 2 was added to the purification column and centrifuged for another 2 min at 12,000× *g*. mRNA from was eluted using RNAse-free water though a RNeasy column. The quality and quantity of mRNA was assessed using NanoDrop 1000. Briefly, 2 uL of RNAse-free water was used to blank the NanoDrop. A total of 2 uL of sample was loaded onto the pedestal and quantified. Quality of mRNA was determined using the 260/280 and 260/230 ratios.

### 2.7. cDNA and Real-time PCR

mRNA (1 µg of RNA/reaction) was reverse-transcribed to cDNA using an Applied Biosystems cDNA synthesis kit (4368814; Fisher Scientific). The quality of cDNA was assessed via spectrophotometry (NanoDrop 1000 Spectrophotometer, Thermo Fisher). Samples of cDNA were diluted to 5 ng/µL (20 µg/reaction) and Radiant^TM^ Green Hi-ROX Green (QS2005; Alkali Scientific, Fort Lauderdale, FL, USA) was utilized for all real-time polymerase chain reaction (qPCR) and was performed on a Step One Plus system (Applied Biosystems). cDNA was activated at 95 °C for 2 min followed by 20 cycles of 95 °C for 5 s (denaturation) and 60 °C for 20 s (annealing/extension). Quantitative real-time qPCR experiments assessed genes involved in cell cycle regulation and pro-apoptotic markers including Cyclin D, cyclin dependent kinase 4 (CDK4), protein 27 (p27), protein 21 (p21), and Hairy and Enhancer of Split 1 (HES1) (Table 1). Glyceraldehyde 3-phosphate (GAPDH) was the housekeeping gene for all qPCR experiments. 

### 2.8. Western Blotting

Western blotting was used to assess proteins regulating cell growth and metabolism. Protein samples prepared in 1x loading buffer, supplemented with 10% β-mercaptoethanol, were denatured at 95 °C for 3 min and then immediately placed on ice for 5 min. Protein samples (30 µg/well) were loaded onto 8% or 10% SDS-page gels and were run at 225 V for 40 min for 8% gels and 43 min for 10% gels in 1x running buffer. Following electrophoresis, the gel was placed into 1x Towbin’s transfer buffer, supplemented with 20% methanol, for 15 min. Proteins were transferred onto a 0.45 µM polyvinylidene difluoride (PVDF-FL) membrane at 100 V for 90 min at 4 °C. Following transfer, membranes were washed once in 1x Tris-buffered saline (TBS) for 5 min. Next the membrane underwent blocking in Odyssey Blocking Buffer and TBS (1:1) for 1 h at room temp. After blocking, the primary antibodies were added overnight (16 h). Primary antibodies included: mammalian target of rapamycin (mTOR), regulated in development and DNA damage responses 1 (REDD1), 5′ Adenosine monophosphate-activated protein kinase (AMPK), Protein Kinase B (PKB/AKT), tumor suppression include protein 53 (p53), and Signal transducer and activator transcription 3 (STAT3). Following removal of the primary antibodies, the membrane underwent 3 × 5 min washes in 1x Tris-buffered saline with Tween 20 (TBST). Secondary antibodies (1:100,000 in TBST) were targeted to primary antibodies and incubated at room temp for 2 h. Next, membranes were washed twice in 1x TBST and twice in 1x TBS. Membranes were imaged using the Odyssey^®^ Licor CLx System. Using the same software, bands were quantified using arbitrary units as a measure of integrated optical density. Phosphorylation (pSTAT3, pAKT, pmTOR, pAMPK, p-p53) proteins were normalized to total (STAT3, AKT, mTOR, AMPK, p53) protein expression. Total (STAT3, AKT, mTOR, AMPK, REDD1, p53) proteins were normalized to Glyceraldehyde 3-phosphate dehydrogenase (GAPDH).

### 2.9. Immunofluorescence Staining

Following 48 h of treatment, cells were washed two times with 1x PBS. Cells were fixed in cold mixture of 70% ethanol and 30% acetone for 10 min at room temperature. Cells were washed two times with 1x PBS. Cells were permeabilized with 0.05% Triton X-100 in 10 min at room temperature. Following another two rounds of washes with 1x PBS, cells underwent blocking with an immunohistochemistry (IHC) blocking buffer for 30 min at room temperature. Next, cells were incubated with a primary antibody for Ki-67 (9129; Cell Signaling, Danvers, MA, USA) diluted in IHC blocking buffer at room temperature for two hours. Cells were washed three times with 1x PBS. Cells were incubated with a AlexaFluo 488 goat-anti-rabbit IgG secondary antibody (A11008; ThermoFisher) for 30 min at room temperature. All cells were washed three more times with 1x PBS. Cells were mounted using 4′,6-diamidino-2-phenylindole (DAPI) Fluoromont-G^®^ (0100-20; SouthernBiotech, Birmingham, Al, USA) and a coverslip.

### 2.10. Immunofluorescence Quantification

Immunofluorescence images were captured using a IX71 inverted fluorescence microscope (Olympus^TM^). Six images per well were captured using a consistent scanning pattern for every well. Separate images were taken for DAPI (International Standards Organization (ISO): 200; exposure 32.05 milliseconds) and Ki-67 (ISO: 400; exposure: 57.87 milliseconds) at their respective wavelength. Background was subtracted (contrast = 8) for all images prior to merging. Co-localization of Ki-67 in nuclei was quantified using FIJI (Binary Feature Extractor). Briefly, merged images were split and brightness and threshold settings were consistent between images for both nuclei detection and sufficient dissection of clustered nuclei. The watershed feature was applied, and the Biovoxxel plugin was used to extract binary features (Objects: DAPI; Selectors; Ki-67).

### 2.11. Statistical Analyses

A two-way ANOVA (time x treatment) was run to detect differences in NSCLC growth following treatment with varying concentrations of metformin (5 mM–15 mM). A Tukey’s post-hoc test was used to further detect differences. All treatments were run with an unpaired *t*-test, except where variances significantly differed (*p* < 0.05). In those cases, a Welch’s *t*-test was used to compare differences in gene expression between treatment groups. Statistical significance was set at an *a priori* alpha value of *p* ≤ 0.05. All statistical analyses and graphs were completed using GraphPad Prism (version 9.1). Data are presented means ± SEM.

## 3. Results

### 3.1. Metformin Inhibited NSCLC Cell Proliferation 

**Figure 1:**
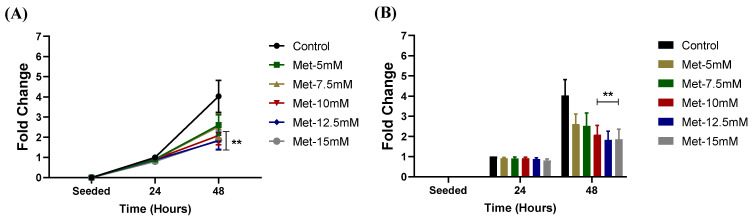
**Proliferation of Lewis lung carcinoma cells following metformin treatment over 48 h.** (**A**,**B**) Fold change (relative to control) in proliferating Lewis lung carcinoma (LL/2) cells treated with 5 mM, 7.5 mM, 10 mM, 12.5 mM, or 15 mM metformin for 24 and 48 h. Data were analyzed using a two-way ANOVA followed by Tukey’s multiple comparison test. ** *p* < 0.01 vs. Con (n = 5). Data are mean ± SEM.

Following incubation with metformin, NSCLC proliferation significantly decreased with both time [*F*(2, 72) = 87.69, *p* < 0.0001] and dose [*F*(5, 72) = 2.364, *p* = 0.0482]. Tukey’s post-hoc test revealed that control cells underwent significantly increased growth over 48 h (*p* < 0.0001). Following a 48 h metformin treatment at 10 to 15 mM, proliferation of NSCLC cells was reduced compared to that of control cells (10 mM metformin, *p* = 0.0094; 12.5 mM metformin, *p* = 0.0015; 15 mM metformin, *p* = 0.0018) (Figure 1). The minimal effective dose of metformin to reduce NSCLC proliferation, i.e., 10 mM, was used in subsequent experiments.

### 3.2. In Vitro NSCLC Cell Gene Expression after a 48 h Metformin Treatment

**Figure 2:**
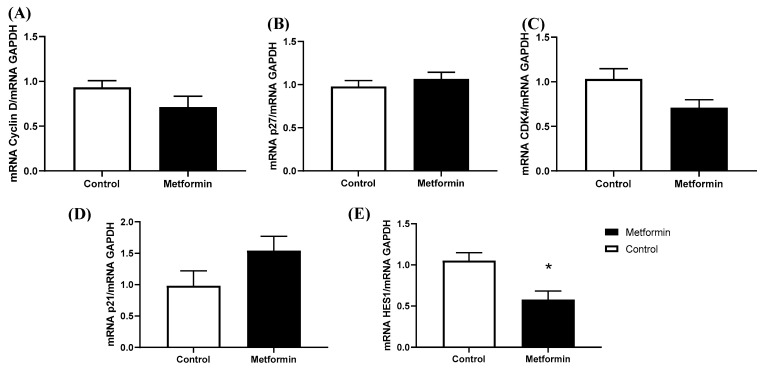
**Gene expression in Lewis lung carcinoma cells treated with 10 mM metformin.** (**A**) mRNA Cyclin D/GAPDH; (**B**) mRNA cyclin dependent kinase inhibitor (p27)/GAPDH; (**C**) Cyclin dependent kinase 4 (CDK4)/GAPDH; (**D**) mRNA cyclin dependent kinase inhibitor (p21)/GAPDH; (**E**) mRNA hairy and enhancer of split 1 (HES1)/GAPDH. mRNA expression in LL/2 cells treated with or without 10 mM metformin. Data were analyzed using an unpaired *t*-test. * *p* = 0.01 vs. Control (n = 5). Data are mean ± SEM.

A total of 10 mM metformin treatment for 48 h did not significantly reduce NSCLC cell gene expression of cell cycle regulators Cyclin D (*p* = 0.166), CDK 4 (*p* = 0.059), P27 (*p* = 0.422), and P21 (*p* = 0.125). However, treatment with 10 mM metformin did significantly reduce NSCLC cell HES1 expression (*p* = 0.011; Figure 2), suggesting HES1 may be involvemed in metformin’s anti-tumorigenic properties. 

### 3.3. In Vitro NSCLC Cell Protein Expression following a 48 h Metformin Treatment

**Figure 3:**
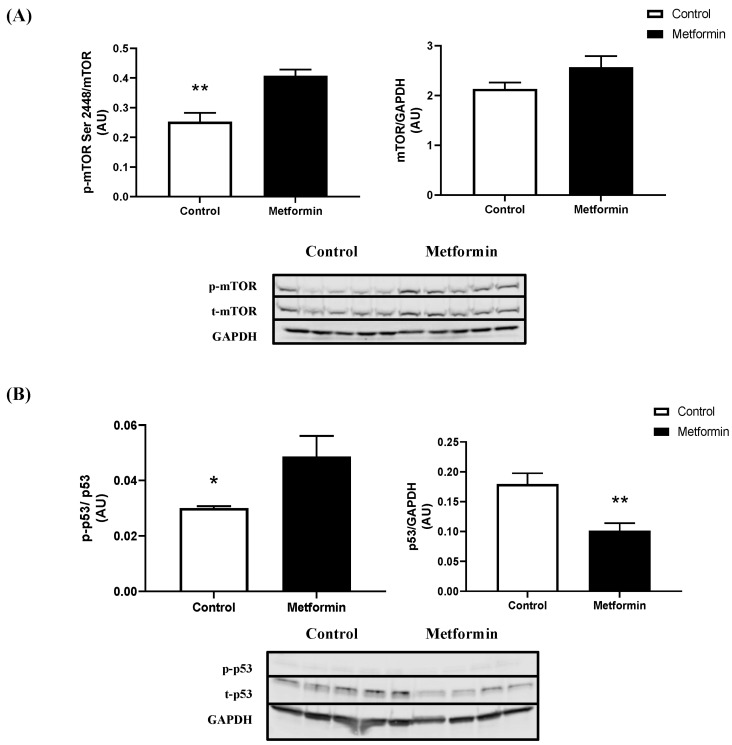
**mTOR and p53 expression in Lewis lung carcinoma cells treated with 10 mM metformin.** (**A**) Phospho (p)- mechanistic target of rapamycin (mTOR)/Total mTOR and mTOR/GAPDH expression (arbitrary units, AU) in LL/2 cells treated with or without 10 mM metformin. Data were analyzed using an unpaired t-test. ** *p* < 0.01 vs. control (n = 5). Data are mean ± SEM. (**B**) Phospho (p)-protein 53(p53)/Total p53 and p53/GAPDH expression (arbitrary units, AU) in LL/2 cells treated with or without 10 mM metformin. Data were analyzed using an unpaired t-test. * *p* < 0.1, ** *p* < 0.01 vs. control (n = 5). Data are mean ± SEM.

**Figure 4:**
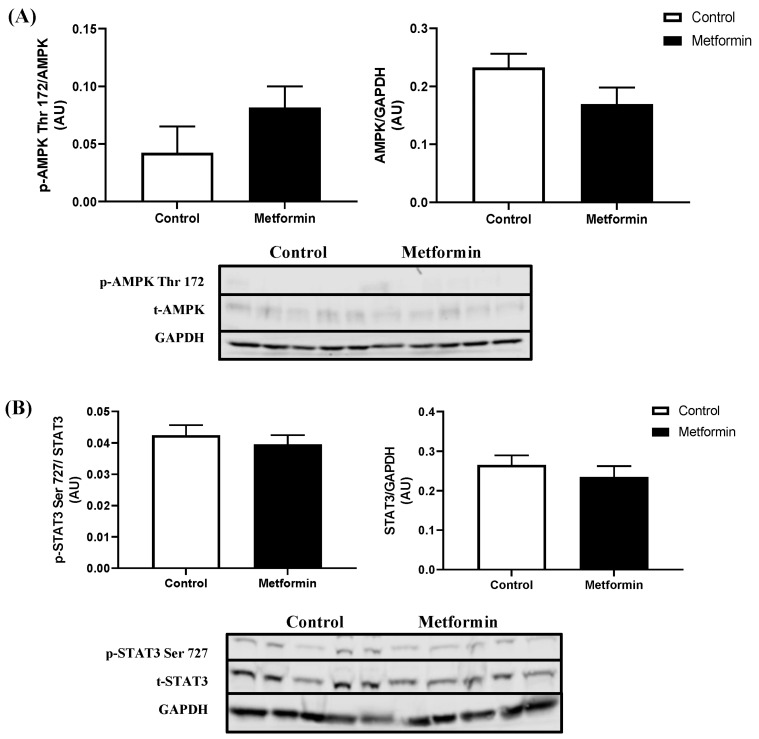
**STAT3 and AMPK expression in Lewis lung carcinoma cells treated with 10 mM Metformin.** (**A**) Phospho (p)- Signal transducer and activator of transcription 3 (STAT3) Ser727/Total STAT3 and STAT3/GAPDH expression (arbitrary units, AU) in LL/2 cells treated with or without 10 mM metformin. Data were analyzed using an unpaired *t*-test. Sample size: n = 5. Data are mean ± SEM. (**B**) Phospho (p)- adenosine monophosphate-activated protein kinase (AMPK) Thr172/Total AMPK and AMPK/GAPDH expression (arbitrary units, AU) in LL/2 cells treated with or without 10 mM metformin. Data were analyzed using an unpaired *t*-test. Sample size: n = 5. Data are mean ± SEM.

**Figure 5:**
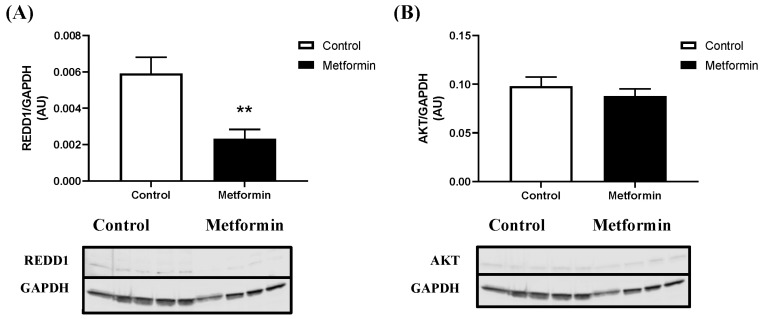
**REDD1 and AKT expression in Lewis lung carcinoma cells treated with 10 mM metformin.** (**A**) Regulated in development and DNA damage responses 1 (REDD1)/GAPDH expression (arbitrary units, AU) in LL/2 cells treated with or without 10 mM metformin. Data were analyzed using an unpaired *t*-test. ** *p* < 0.01 vs. control (n = 5). Data are mean ± SEM. (**B**) Protein kinase B (AKT)/GAPDH expression (arbitrary units, AU) in LL/2 cells treated with or without 10 mM metformin. Data were analyzed using an unpaired *t*-test (*p* = 0.4135). Sample size: n = 5. Data are mean ± SEM.

Metformin increased p-mTOR Ser2448 protein expression in NSCLC cells when compared to control treated cells (*p* = 0.003). Total mTOR protein expression remained the same, irrespective of treatment (*p* = 0.134) (Figure 3). Furthermore, metformin treatment increased the phosphorylated-to-total ratio of p53 (*p* = 0.0367). However, this is likely the result of reduced total p53 expression following metformin treatment. Interestingly, metformin treatment reduced total p53 protein expression (*p* = 0.0078; Figure 3). Metformin did not significantly alter the expression of p-AMPK Thr172 (*p* = 0.2390), total AMPK (*p* = 0.0730; Figure 4), p-STAT3 Ser727 (*p* = 0.5291), or total STAT3 (*p* = 0.4274; Figure 4) protein expression. Metformin treatment significantly decreased REDD1 protein expression (*p* = 0.0082; Figure 5). There were no marked differences in total AKT protein expression (*p* = 0.4135; Figure 5).

### 3.4. Ki-67 Immunofluorescence in NSCLC Cells following a 48 h Metformin Treatment

**Figure 6:**
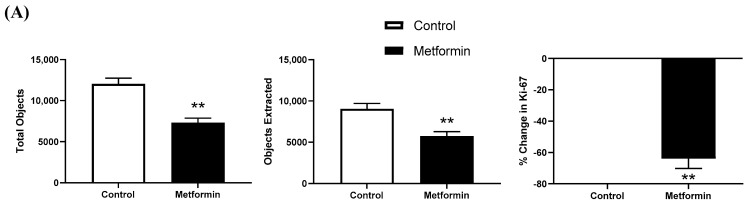

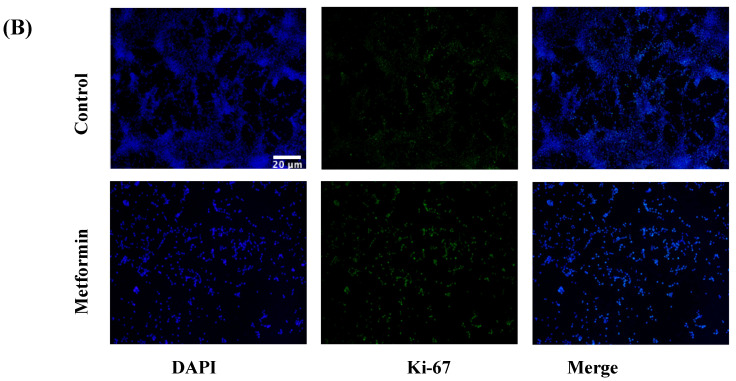
**Ki-67 immunofluorescence in Lewis lung carcinoma cells treated with 10 mM metformin.** (**A**) Total objects and objects extracted using binary feature extractor via Fiji. Percent change in Ki-67 expression relative to control in 48 h LL/2 cells treated with or without 10 mM metformin. ** *p* < 0.01 vs. control (n = 4). Data are mean ± SEM. (**B**) Representative immunofluorescence images of LL/2 cells treated with or without 10 mM metformin using 4′,6-diamidino-2-phenylindole (DAPI) and Ki-67 expression visualized via AlexaFluor 488. Representative images were taken at 10x. Scale bar = 20 µm.

A total of 10 mM metformin treatment for 48 h significantly reduced Ki-67 expression by ~65% in NSCLC cells (*p* = 0.0021). The total number of nuclei was significantly reduced after treatment with 10 mM metformin (*p* = 0.0020). The percent of co-localized Ki-67 did not differ between treatments (*p* = 0.4671; Figure 6). 

### 3.5. Ki-67 Immunofluorescence in NSCLC Cells after a 48 h GSI Treatment

**Figure 7:**
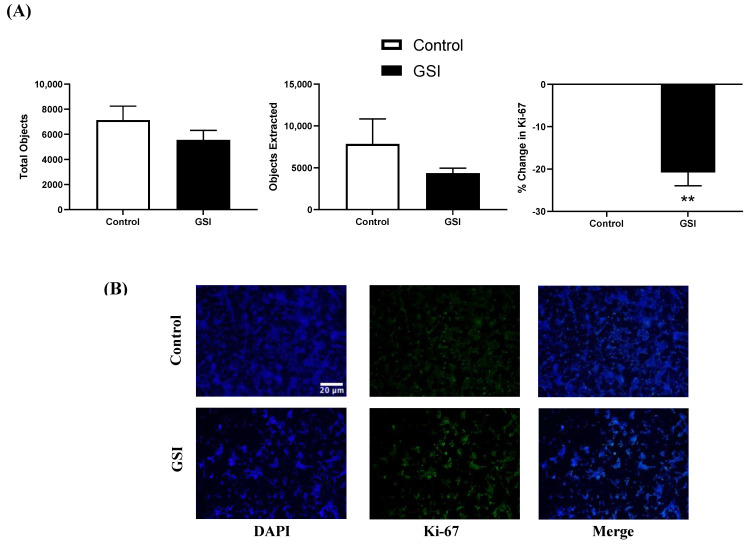
**Ki-67 immunofluorescence in Lewis lung carcinoma cells treated with GSI.** (**A**) Total objects and objects extracted using binary feature extractor via Fiji. Percent change in Ki-67 expression relative to control in 48 h LL/2 cells treated with or without 4 µM Ɣ-secretase inhibitor (GSI) every 12 h. ** *p* < 0.01 vs. control (n = 5). (**B**) Representative immunofluorescence images of LL/2 cells treated with or without 10 mM metformin using 4′,6-diamidino-2-phenylindole (DAPI) and Ki-67 expression visualized via AlexaFluor 488. Representative images were taken at 10x. Scale bar = 20 µm.

In Figure 2 we reported that metformin reduced HES1 gene expression. HES1 is a downstream target of notch signaling, and the overactivation of notch signaling has been documented as a contributor to NSCLC tumorigenesis [27,28]. Therefore, we sought to determine whether inhibiting notch with a gamma-secretase inhibitor (GSI) will suppress Ki-67 expression. A 48 h GSI (4 µM) treatment of NSCLC cells significantly reduced Ki-67 expression (~20%, *p* = 0.0028). However, the total number of nuclei and Ki-67 extracted were not significantly different with Ɣ-secretase inhibition (*p* = 0.2716 and *p* = 0.6473, respectively; Figure 7).

### 3.6. Ki-67 Immunofluorescence in NSCLC Cells following a 48 h Co-Treatment with GSI and Metformin

**Figure 8:**
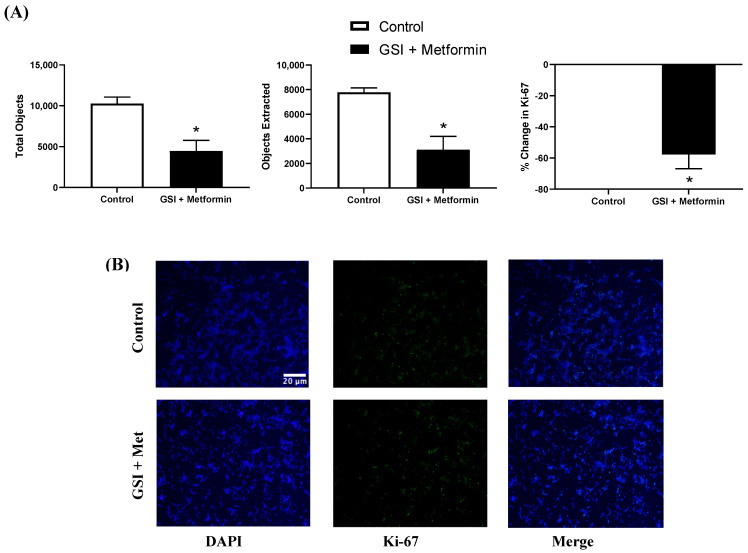
**Ki-67 immunofluorescence in Lewis lung carcinoma cells treated with GSI and 10 mM metformin.** (**A**) Total objects and objects extracted using binary feature extractor via Fiji. Percent change in Ki-67 expression relative to control in 48 h LL/2 cells treated with or without 10 mM metformin and 4 µM Ɣ-secretase inhibitor (GSI) every 12 h. * *p* = 0.02 vs. control (n = 3). (**B**) Representative immunofluorescence images of LL/2 cells treated with or without 10 mM metformin using 4′,6-diamidino-2-phenylindole (DAPI) and Ki-67 expression visualized via AlexaFluor 488. Representative images were taken at 10x. Scale bar = 20 µm.

A 48 h co-treatment with 4 µM GSI and 10 mM metformin significantly reduced Ki-67 expression by NSCLC cells (>50%; *p* = 0.0245). The total number of nuclei was also significantly reduced after a 48 h co-treatment with metformin (10 mM) and GSI (4 µM) (*p* = 0.0191). However, the percent of extracted Ki-67 did not differ between treatments (*p* = 0.1969; Figure 8). 

## 4. Discussion

The present study aimed to determine the effects of metformin on LL/2 lung cell growth in vitro. These findings indicate that 48 h of metformin exposure (≥10 mM) decreases NSCLC growth in vitro, evidenced by decreased proliferation. Metformin treatment (≥10 mM) increased the phosphorylated-to-total-p53 ratio and decreased total p53 protein, suggesting cell cycle arrest driving the reduction in proliferation. Metformin treatment (≥10 mM) staunchly reduced HES1 expression, which is regulated by Notch, Wnt, or Hedgehog signaling, so this alteration in gene expression could be an important moderator of NSCLC [29]. Metformin treatment (≥10 mM) also reduces p-mTOR Ser 2448; however, total mTOR protein expression remains similar irrespective of treatment, suggesting that metformin exposure as a monotherapy (≥10 mM) is not sufficient to completely abrogate cell growth. Our findings are supported by previous literature showing that metformin (5 µM–20 mM) does decrease NSCLC cancer cell proliferation [30,31]. Lower doses of metformin (5 µM–5 mM) have been shown to inhibit human NSCLC cell proliferation in vitro, in a dose-dependent manner [31]. However, the data obtained highlighted that concentrations below 10 mM did not reduce proliferation when compared to control over 48 h of treatment, suggesting that 10 mM metformin was the lowest effective does to combat progression of this Lewis lung cancer cell line. Differences in metformin sensitivity are likely cell-line-dependent, with many studies using human cell lines, whereas the present study used a murine cell line derived from a C57BL mouse. Metformin concentrations lower than 10 mM, i.e., mirrored pharmacological dosing seen in the clinical population, were ineffective at preventing LL/2 Lewis lung cell proliferation in vitro. Thus, the metformin sensitivity of LL/2 cells may be challenging to translate to human therapy, as higher doses (1–50 mM in vitro) lead to in vivo concentrations that are not safely achievable or relevant to the clinical population [31,32].

Despite significantly reduced proliferation, gene expression of key cell cycle regulators (p27, p21, CDK4, Cyclin D) within the LL/2 tumor cells were not significantly altered by metformin. These markers were chosen because p27 and p21 can act as tumor suppressors and inhibit cell cycle progression [33,34,35]. CDK4 and Cyclin D are key players controlling regulation of the G1-S checkpoint and often undergo dysregulation in cancer cells [36]. Previous studies showed that human lung cancer cell lines treated with metformin (10–40 mM) do undergo anti-neoplastic effects, evidenced by repressing cell growth and cell cycle regulatory proteins, namely p27, p57, and PTEN [37]. Metformin (20 mM) induces cell cycle arrest, reduced proliferation but not apoptosis in ovarian cancer cell lines [38]. Breast cancer cells do show increased p27 expression when treated with metformin (10 mM) for 48 h [10]. However, most of these effects again were noted in response to much higher doses of metformin treatment (≥10 mM) in vitro. 

While no marked changes were detected in these cell cycle regulatory genes, HES1 showed significant reductions in response to metformin treatment (10 mM). Because HES1 expression decreased when LL/2 cells were treated with metformin, NSCLC growth may have been at least partially mediated through Notch signaling. To date, our data provide the first evidence of metformin’s efficacy on NSCLC cell lines may involve HES1 expression. However, colorectal cancer patients with co-morbid type 2 diabetes have abnormal cellular proliferation, which was correlated with increased Notch1/HES1 signaling and curbed by metformin treatment [39]. Notch1 is a potential independent prognostic factor for small cell lung carcinoma patients (n = 46), with a small cohort having a high expression of Notch-1 (n = 10) [40]. In addition to its role in cell cycle regulation, cellular growth, and survival, HES1 is also regulated through other pathways besides Notch (i.e., Wnt and Hedgehog signaling pathways) and is a key player in T cell development [29]. Metformin may target cancers through HES1 signaling; however, much surrounding this potential mechanism of action still requires further study.

As an AMPK activator, metformin would be expected to act along its canonical mechanism of action, resulting in slowed growth through LKB-1 activation of AMPK [15]. Activated AMPK also induces cell cycle arrest through activation of protein 53 (p53), leading to upregulation of pro-apoptotic genes [17,18]. However, the present findings revealed no detectable differences in p-AMPK or total AMPK protein expression in LL/2 cells treated with metformin (10 mM) for 48 h. However, alterations in p53 were present. Specifically, metformin treatment increased the ratio of phosphorylated to total p53 protein while reducing total p53 protein expression. This could be indicative of a metformin-induced downregulation of total p53 protein. Previous studies have shown metformin (20 mM) induced alterations in metabolism, including AMPK and glycolysis in ovarian cancer cell lines [38]. Further studies combining metformin treatment (4–16 mM) with low-dose celecoxib (an anti-inflammatory drug, 4–16 µM) show staunch reduction in NSCLC cell migration, invasions, and increased expression of p53, resulting in cell cycle arrest [41]. In breast cancer cells, AMPK has also been shown to activate forkhead transcription factors (FOXO), a protein family that can act as a tumor suppressor through promotion of cell cycle arrest, DNA damage repair, and apoptosis [10]. Metformin does elicit tumor-suppressing properties, particularly via p53 in this LL/2 cell line; however, other published studies better support the activation of AMPK via metformin and downstream repression of tumor growth. 

The Lewis lung carcinoma mouse model has attenuated expression of fundamental genes involved in the phosphatidylinositol 3-kinase (PI3K)–protein kinase B (Akt) pathway [22]. The PI3K/AKT pathway plays an important role in cellular proliferation, growth, metabolism, and protein synthesis and is often constitutively active in tumor cells [23]. Regulated in development and DNA damage response (REDD1) is a ubiquitous protein that is a well-known endogenous inhibitor of the AKT/mTOR pathway [42]. Thus, not surprisingly, REDD1 does play a role in regulating cell growth, mitochondrial function, oxidative stress, and apoptosis [43]. In the present study, metformin treatment (10 mM) significantly reduced REDD1 expression in LL/2 cells, but overall AKT expression remained similar between both control and metformin-treated cells. If REDD1 inhibited mTOR activity, its expression would be expected to increase following metformin treatment. In contrast to the present study, REDD1 expression inhibits NSCLC invasiveness in human cell lines via mTOR suppression [44]. Prostate cancer cells treated with metformin showed increased expression of REDD1, promoting mTOR inhibition and cell cycle arrest [45]. Our findings in LL/2 differ from prior observations demonstrating a role for REDD1 in tumor suppression. A better understanding of the role of REDD1 negatively regulating mTOR activity in LL/2 cells may be obtained through investigating REDD1 expression following treatment combining metformin with an mTOR inhibitor or a targeted chemotherapeutic.

Metformin treatment (10 mM) over 48 h reduced p-mTOR Ser2448 but not total mTOR protein in control NSCLC cells. These findings are partially supported by another study of lung cancer cells exposed to metformin over 72 h [46]. Metformin treatment (10 mM), particularly in combination with rapamycin (an inhibitor of mTOR), reduced the viability of human lung cancer cells that were resistant to cisplatin [46]. Proteomic analyses revealed that these anti-proliferative effects were associated with mTOR signaling [46]. In lung cancer cells, metformin was more effective as a co-treatment promoting then the most anti-proliferative effect. 

STAT3 also plays a critical role, not only in signal transduction, but also through cancer-promoting inflammation and increasing anti-tumor immunity [47]. In human NSCLC, metformin targets STAT3, curbing tumor proliferation [48]. Surprisingly, our present findings revealed no change in phosphorylation or total STAT3 expression. Indeed, STAT3 is often upregulated in many cancers. In particular, metformin inhibits cell growth, through targeting STAT3, in triple-negative breast cancers [49]. Normally these cancers show upregulation of STAT3 and phosphorylation of STAT3 at Tyr705 and Ser727 and are partially sensitive to metformin, promoting a strong growth-inhibitory and apoptotic effect in these cells [49]. In addition to breast cancers, metformin treatment (125 mg/kg) suppresses pancreatic tumor growth in genetically engineered mice, evidenced by lower tumor volume at the end of the study (1 or 3 weeks) [12]. Metformin treatment decreased phosphorylation at both STAT3 and NF-κB [12].

Immunofluorescence imaging showed that metformin treatment significantly reduced total nuclei and Ki-67 expression with a >65% reduction for the latter between control and metformin-treated cells. Therefore, metformin treatment (10 mM for 48 h) slowed growth of LL/2 cells without abrogating growth. Ki-67 expression correlates with metastasis and tumor stage in clinical populations [50]. The metformin-reduced proliferation was further assessed when combined with a γ-secretase inhibitor (GSI) preventing the cleavage of γ-secretase used to impede downstream effects of mTOR in an effort to better understand the potential role of mTOR regulation in proliferation of LL/2 cells. In the conditions tested, GSI treatment alone did not have as stringent of an effect on the LL/2 cells. Indeed, staining revealed ~20% reduction in Ki-67 proliferation, which is auspicious but nominal compared to metformin alone. The treatment combination of metformin and GSI led to reduced proliferation and ~50% reduction in Ki-67 expression, although no complete abrogation was observed. This could suggest that growth of NSCLC acts independently of Notch. Since inhibition of γ-secretase, a key enzyme in Notch activation, did not completely stop growth of LL/2 cells, LL/2 lung cancer cell growth is probably not solely regulated via Notch. While inhibiting Notch signaling is effective, it is not the most effective treatment for these cells. Combining metformin and GSI resulted in a similar effect—significantly reduced growth—rather than a dramatic additive effect.

## 5. Conclusions

Based off the present study, metformin treatment (10 mM for 48 h) alone is not sufficient to completely abrogate LL/2 cell growth in vitro. The reduction in proliferation via Ki-67 suggests that metformin treatment (10 mM for 48 h) does have a significant anti-proliferative effect but does not elicit many marked effects when it comes to genes regulating cell cycle progression or proteins involved with cell growth, proliferation, and metabolism. Importantly, since GSI did not fully blunt NSCLC proliferation, it is highly probable that NSCLC growth is not solely regulated via Notch signaling. Metformin (10 mM for 48 h) is an effective pharmaceutical to reduce cancer cell proliferation when directly added to LL/2 cells in vitro, but the potential role of metformin as an anti-cancer intervention for patients with NSCLC requires further study and may be most beneficial if metformin is used as part of multi-therapies. This is supported by recent in vivo work demonstrating that metformin alone is not sufficient to reduce NSCLC tumor burden [51]. Indeed, metformin was more effective when combined with another treatment modality (i.e., ionizing radiation, chemotherapeutic, or inhibitor) [31,46,52,53]. Future studies should be conducted to further investigate metformin as a co-therapy and approached with more targeted drug delivery tactics.

## Figures and Tables

**Table 1:** Primers used for gene expression analyses.

Primer		Sequence
Cyclin D	Forward	GATGGCGATCGTCCTGTCAT
	Reverse	ACAGGCCGCTACAAGAAACA
CDK4	Forward	ATGGCTGCCACTCGATATGAA
	Reverse	TCCTCCATTAGGAACTCTCACAC
p27	Forward	TCTCTTCGGCCCGGTCAAT
	Reverse	AAATTCCACTTGCGCTGACTC
p21	Forward	TGGTGATGTCCGACCTGTT
	Reverse	CATGAGCGCATCGCAATC
HES1	Forward	GGTCCTGGAATAGTGCTACCG
	Reverse	CACCGGGGAGGAGGAATTTTT
GAPDH	Forward	ATGTTTGTGATGGGTGTGAA
	Reverse	ATGCCAAAGTTGTCATGGAT

CDK4: Cyclin dependent kinase 4; p27: cyclin dependent kinase inhibitor protein 27; p21: cyclin dependent kinase inhibitor protein 21; HES1: Hairy and enhancer split protein; GAPDH: Glyceraldehyde 3-Phosphate Dehydrogenase.

## Data Availability

For access to available data, please contact the corresponding author.
